# Structural Variant Detection by Large-scale Sequencing Reveals New Evolutionary Evidence on Breed Divergence between Chinese and European Pigs

**DOI:** 10.1038/srep18501

**Published:** 2016-01-05

**Authors:** Pengju Zhao, Junhui Li, Huimin Kang, Haifei Wang, Ziyao Fan, Zongjun Yin, Jiafu Wang, Qin Zhang, Zhiquan Wang, Jian-Feng Liu

**Affiliations:** 1 National Engineering Laboratory for Animal Breeding; Key Laboratory of Animal Genetics, Breeding and Reproduction, Ministry of Agriculture; College of Animal Science and Technology, China Agricultural University, Beijing, 100193, China; 2 College of Animal Science and Technology, Anhui Agricultural University, Hefei, 230036, China; 3 School of Animal Science, Guizhou University, Guiyang, 550025, China; 4 Department of Agricultural, Food & Nutritional Science, University of Alberta, Edmonton, T6G 2C8, Canada

## Abstract

In this study, we performed a genome-wide SV detection among the genomes of thirteen pigs from diverse Chinese and European originated breeds by next genetation sequencing, and constrcuted a single-nucleotide resolution map involving 56,930 putative SVs. We firstly identified a SV hotspot spanning 35 Mb region on the X chromosome specifically in the genomes of Chinese originated individuals. Further scrutinizing this region by large-scale sequencing data of extra 111 individuals, we obtained the confirmatory evidence on our initial finding. Moreover, thirty five SV-related genes within the hotspot region, being of importance for reproduction ability, rendered significant different evolution rates between Chinese and European originated breeds. The SV hotspot identified herein offers a novel evidence for assessing phylogenetic relationships, as well as likely explains the genetic difference of corresponding phenotypes and features, among Chinese and European pig breeds. Furthermore, we employed various SVs to infer genetic structure of individuls surveyed. We found SVs can clearly detect the difference of genetic background among individuals. This clues us that genome-wide SVs can capture majority of geneic variation and be applied into cladistic analyses. Characterizing whole genome SVs demonstrated that SVs are significantly enriched/depleted with various genomic features.

Structural variants (SVs) describe genomic rearrangements that can facilitate adaptation^1^ and speciation^2^,^3^, and are often involved in genetic disorders^4^. As an alternative genetic variation different from single nucleotide polymorphisms (SNPs), SVs involve dosage-altering variations such as insertions and deletions, and dosage-invariant rearrangements such as inversions and translocations^5^. Those kilo base-sized to mega base-sized deletions and insertions are often classified as copy number variations (CNVs), while smaller insertions or deletions (<50 bp) are classified as indels^6^. It has been established that SVs contain more nucleotides than SNPs, and are more significantly related to phenotypic differences^7^,^8^,^9^. It was reported that the contribution of SVs to complex phenotypes has been assessed at gene expression levels, and SVs and SNPs have been manifested accounting for 83.6 and 17.7 percent of total genetic variation, respectively^10^.

So far, a number of researches have been conducted to identify and annotate comprehensive SVs for promoting evolutionary genetic and functional genomic studies in human genomes. Especially, the well-developed next generation sequencing (NGS) technologies allow the study of SVs in basically any organisms. NGS techniques coupled with corresponding analytical algorithms have been developed and successfully applied in construction of SV maps in human^4^ as well as other species, *e.g.*, mouse^11^, dog^12^ and *Drosophila melanogaster*^13^. A more recent genomic SV investigation on African cichlid^14^ proves the role of SVs in the diversification and speciation of organisms.

Given the fact that pigs are the last species sharing a common ancestor with humans^15^, they have been considered as a very popular biomedical model for various human diseases^16^. Theoretically and logically, SVs can be speculated having similar effects in evolution and genetic determination of many aspects of complex phenotypes in pig genomes as those in human genomes. However, studies on SVs, especially those non-CNV SVs in pig genomes, have not received attentions as sufficient as in human genomes to date.

It is well known that Chinese indigenous pig breeds represent one of the most valuable genetic resources in the world, having pronounced differences in domestication and breed formation history with the European pig populations. This has been widely supported by the studies based on the traditional genetic markers of Microsatellite^17^ and SNPs^15^,^18^,^19^. However, the genetic variation across populations could be impossibly captured by merely a single type of markers, which is often reflected by the issue of missing heritability^20^. As an alternative genomic variant different from traditional genetic markers, it is still unclear if SVs contribute to the phylogeny of different populations as well as genetic diversity of phenotypes.

In this context, to explore the role of SVs in pig phylogenetic and breed diversity among diverse populations, we initially performed this study to exploit and to characterize genome-wide SVs among diverse populations originated from both China and Europe. Our findings can broaden the spectrum of pig genomic variation, further facilitating comparative genomic studies between pigs and humans. Besides, to pursue an in-depth understanding of genomic structure and conformation, we further infer mechanisms of SV formation and compare the relationship between SVs with various pig genomic features.

To obtain highly convincing SV detections, we employed a suite of most-commonly used methods based on high throughput NGS data generated from the genomes of 13 pig individuals in SV callings. These 13 individuals were intentionally selected from ten distinct pig breeds with distant genetic backgrounds between each other. For further confirming the population-specific SV regions underlying genetic diversity across different populations, we additionally employed large-scale NGS data of 111 individuals originated from both Chinese and European populations in the follow-up analyses. This further supported our findings on the role of SV in pig evolution.

We expected that our findings of SVs across pig genomes would provide novel evidence for pig phylogenetic studies, which could also facilitate SV functional analyses and other SV-related genomic studies in the future.

## Results

### Paired-end sequencing and mapping patterns

Based on the sequencing data set, we calculated the depth of coverage for each animal, which varied from 10.4 (a Duroc pig) to 17.4 (a Rongchang pig) folds with the average of 11.95. Accordingly, the average of sequence coverage reached 98.41% and the average mapping count per read was 1.15 based on the paired-end reads. Obviously, the raw data set passed our sequence quality filters, reaching the criteria for follow-up genome-wide SV detections.

### Genome-wide SV identification

**Figure 1:**
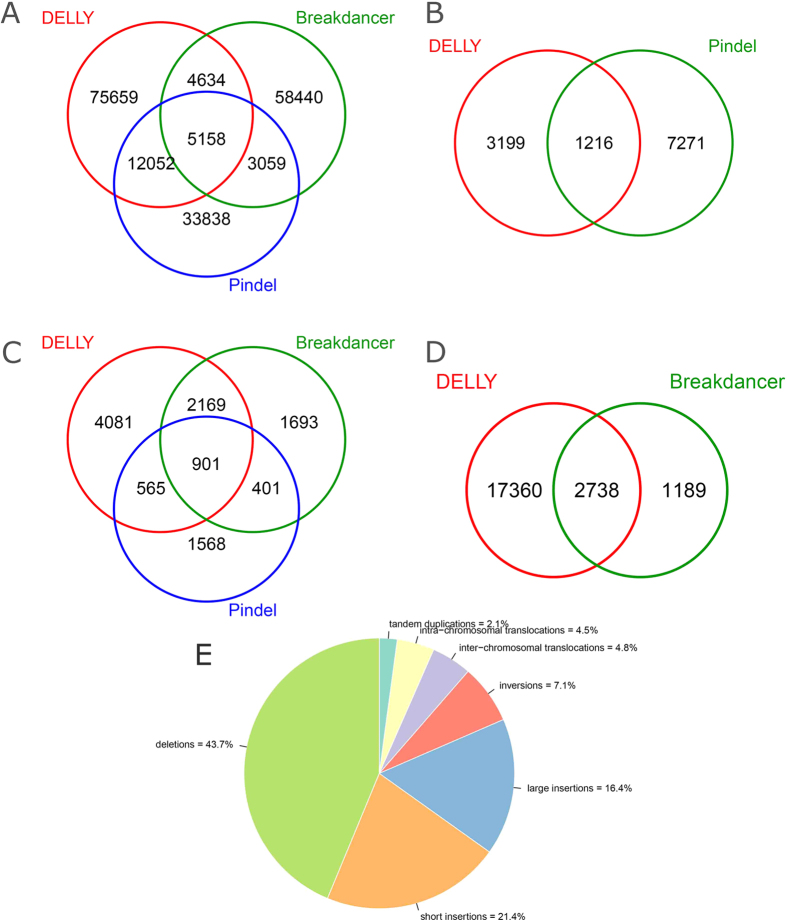
Venn diagram for the detection software and proportion of different SVs. (**A**) Venn diagram depicts the proportion of deletions detected based on DELLY, Breakdancer, and Pindel, respectively. (**B**) Venn diagram depicts the proportion of tandem duplications detected based on DELLY and Pindel, respectively. (**C**) Venn diagram depicts the proportion of inversions detected based on DELLY, Breakdancer, and Pindel, respectively. (**D**) Venn diagram depicts the proportion of inter-chromosome translocations detected based on DELLY and Breakdancer, respectively. (**E**) The pie chart includes all types of SVs, in which each slice of the pie represents one type of SV.

Following the definition in previous report^21^, the alleged calling SVs in this study mainly refer to common SVs larger than 50 bp in size, which included all deletions and inversions, tandem duplications, intra-chromosomal translocations, and inter-chromosomal translocations. The sequencing data that passed quality control and recalibration were subsequently processed by Breakdancer^22^, Pindel^23^ and DELLY^24^ for calling SVs. The three detection tools employed herein are based on different strategies of read pair (RP), Split Read (SR) and a combination of RP and SR, respectively. Besides, for removing the gender effect on SVs detection, the Y chromosome was excluded from our analyses. Figure 1 gave a general profile of detections for each type of SVs. In order to obtain more convincing outcomes, we only determined those SVs commonly detected by more than two different software (except translocations and the large insertions).

We eventually identified a total number of 56,930 distinct SVs with a unique position in Sscrofa10.2 as given in Table 1. The detailed information on the different patterns of SVs identified was presented in Table S1,S2,S3,S4,S5,S6–S7. To visually compare genomic distribution of each type of SV among all experimental individuals, we also constructed a comprehensive SV map (See Figure S1). Among these identified SVs, 7,606 (13.7%) were merely found in the genome of a single individual; 11,038 (19.4%) were observed in the genomes of all individual investigated, which may be due to inherent genetic variation, or the imperfect assembly in the reference genome. Besides, 38,286 (67%) out of them were detected with polymorphism among the experimental individuals. We also noticed that the highest number of SVs was in the wild boar individual while the lowest number in the Duroc individual. This could be explained by the substantial structural differences among the genomes of various pig breeds, as well as Chinese wild boars conferring much higher genetic variability in the genome compared with domestic pigs^15^,^19^.

We also observed that the estimate of SVs was far less than that of SNPs (a total of 28.1 million were identified in the genomes of the 13 individuals). This conservative mutation rate of SVs could maintain the fundamental function of the pig genome since SVs have been considered having potentially larger effects than SNPs via changing gene structure and dosage, alternating gene regulation, exposing recessive alleles and other mechanisms^25^,^26^. Furthermore, the most common type of identified SVs was deletions, which is consistent with similar studies in other species^27^. This is because the current SV detection method was prone to calling deletion in contrast to other types of SVs^5^,^24^,^28^.

**Figure 2:**
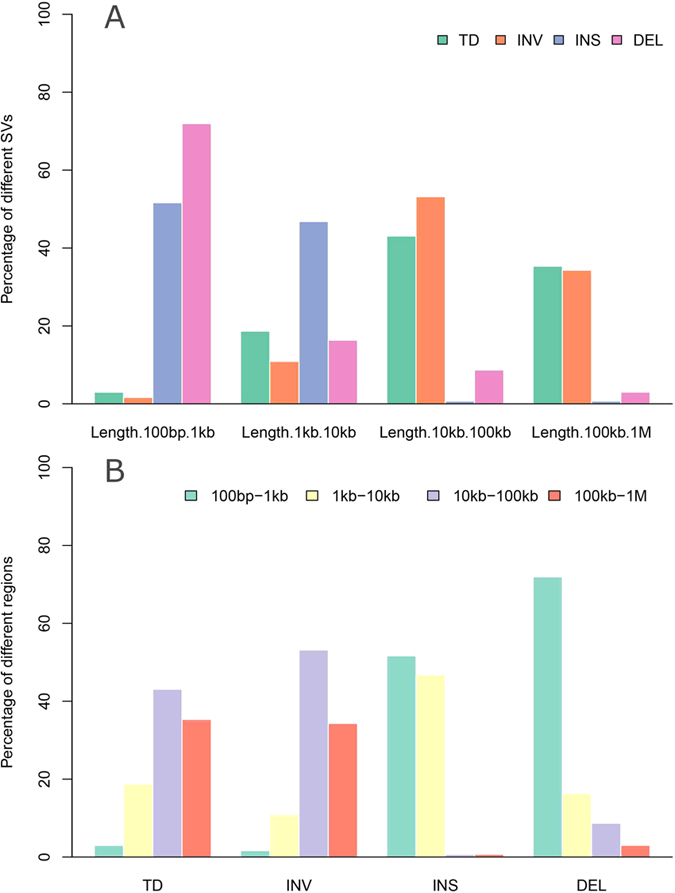
Size distribution of diverse SVs across the genome. (**A**) The percentage of different SVs in the specific SV size ranges. These ranges respectively represent the different order of magnitude for the sizes of the structural variant (100 bp to 1 Mb), the color bar represents the different SV type including tandem duplication (TD), inversion (INV), insertion (INS), and deletion (DEL). (**B**) The percentage of the different SV size ranges in the specific SV, the specific ranges of SV size and the specific color bars also equivalent to (**A**).

We further investigated the size distribution of the identified SVs with the length between 50 bp and 1 Mb across the pig genomes, more than 70% of deletions fell into the length interval from 100 bp to 1 kb, accounting for the majority of SVs with small size of less than 1 kb (Fig. 2). This feature has also been found in the chicken genome^29^; On the contrary, most of inversions (87.46%) ranged from 10 kb to1 Mb in length, spanning the largest region among all types of SVs.

Further comparing the distribution of translocations among different chromosomes, we found that most of the intra-chromosomal translocations rose from Chromosome 1 (Table S8), and the top two incidences of inter-chromosomal translocations occurred between Chromosomes 1 and 2 as well as Chromosomes 1 and 13 (Table S9). This indicated that Chromosome 1 could be the hotspot of translocations among the pig genome.

### Inferring mechanisms of deletion formation

SV formation mechanisms and the bias in SV formation can enhance our understanding of the genome features and facilitate further in-depth studies on effects of genomic variants on phenotypes. At present, the commonly recognized mechanisms of SV formation are mainly including nonallelic homologous recombination (NAHR)^30^, Nonhomologous end joining (NHEJ)^31^, alternative end joining (Alt -EJ)^32^, also known as microhomology-mediated end joining (MMEJ)^33^, variable number of tandem repeat (VNTR)^34^, transposable element insertions (TEIs)^35^ involving mostly long and short interspersed elements (LINEs^36^ and SINEs^37^) and combinations thereof, along with other types of TEI-associated events, and fork stalling and template switching (FoSTeS) or microhomology-mediated break-induced repair (MMBIR)^38^.

**Figure 3:**
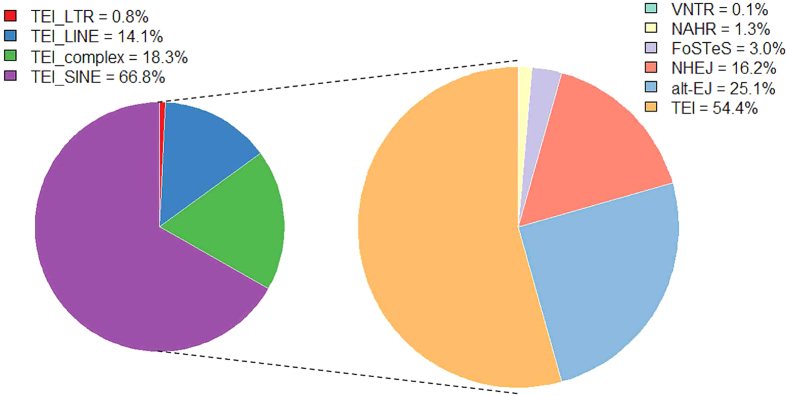
The proportion of different deletion formation mechanisms. The right panel represents relative proportion of deletion formation mechanisms (VNTR, NAHR, FoSTeS, NHEJ, alt-EJ, TEL) observed in all individuals, and left panel represents relative proportion of various TEI types in all TEI formation mechanisms (LTR, LINE, SINE, complex).

We processed a Meerkat pipeline^39^ to infer mechanisms of the deletion formation. Among all 24,903 identified deletions, 18,796 out of them (75.5%) were successfully categorized into six subsets according to their inferred formation mechanisms (Fig. 3). It is clear that the TEI was the dominating formation mechanism for deletion events, accounting for 54.4% of all deletions analyzed. The similar feature of deletion mechanisms was also found in pig^19^, humans^40^, chimpanzee, orang-utan, and rhesus macaque^41^. Further classification of TEI events showed that majority of TEIs is determined as SINEs, which are much more than LINEs and LTR-elements. This may be due to that SINEs have relatively short size and more likely neutral compared to others. The feature of this inferred mechanism has also been reported in other eukaryotic genome previously, such as human^37^ and rice^42^.

Additionally, among all those deletions inferred as the mechanism of TEIs in our study, approximately 66.8% out of them belong to SINEs (Fig. 3), demonstrating SINEs play a key role in the formation of deletion variants. This feature has been clearly proven by Ai *et al.*^19^, where structural variations and SINE/tRNA elements were found having similar distribution patterns, *i.e.*, more than 50% of structural variations overlapped with SINE/tRNA elements in the genome, supporting that SINE/tRNA elements was an important source of genetic diversity in pigs.

Finally, among all identified deletions, we also detected 1,869 complex SV regions (Table S10) where multiple deletions, duplications or rearrangements occurred at a single locus^43^,^44^ or some small scale SVs or rearrangements happened at the breakpoint of a larger SV^45^,^46^. Among these complex SVs, 53 out of them can be definitely assigned to six kinds of complex deletions illustrated in Figure S2 as well as one type of complex SV that deletion with insertion at the breakpoint with unknown source. Figure S2 has given each of combinations of distinct SV events detected in our studies. All these 53 deletion-related complex SVs as well as their inferred formation mechanism were given in Table 2.

### Refining SV breakpoints via Local *de novo* assembly

We processed a local *de novo* assembly pipeline (Figure S3) to refine SVs breakpoints for pursuing a high accuracy SV map. In our study, the local *de novo* assembly was conducted merely among those SV findings including 49,075 deletions (<65 kb), 27,595 short insertions, 7,365 inversions (<31 kb), 2,246 tandem duplications (<65 kb) and all 106,515 translocations. Finally, we choose the SV breakpoints from the local *de novo* assembly that occurred most frequently and closest to the original breakpoints as the refined breakpoints for each SV in the analyses.

Specifically, for all deletions, we observed relatively accurate breakpoints (bias <50 bp) of 29,015 deletions (59.1%), 14,350 out of which have the perfect overlapping breakpoints (bias = 0 bp) with our original findings, further confirmed the high accuracy outcomes for the identified deletions. Similarly, 14,642 insertions (53%) had relatively accurate breakpoints, 779 of which were perfect overlapping breakpoints. For inversions and tandem duplications, the refined SVs largely had less sizes than their original findings. This might be because that inversions and tandem duplications entailed higher breakpoint complexity compared to that of insertions and deletions, and the limitation of local *de novo* assembly for the relatively large size SVs.

Regarding translocations, in order to reduce the false positives caused by the similarity sequence that from different chromosome regions, we picked up two sequences near corresponding breakpoints from reference to align with each other. Consequentially, only those translocations that had a lower match with reference sequences while with a higher match with the *de novo* assembly sequence were selected as our final translocation outcomes. Based on this strategy, we not only verified the accuracy of translocations, but also predicted direction of translocation and proportion of complex translocation, *i.e.*, transposition and reciprocal translocation. Transposition was a fragment sequence from one chromosome transferred to another chromosome^47^, while the reciprocal translocation was usually an exchange of material between nonhomologous chromosomes^48^. Accordingly, we totally confirmed 8,034 transpositions and 470 reciprocal translocations (Table S11), and it would promote a deep understanding of pig genome structure.

### SV validation through PCR

Additionally, to verify the accuracy of our final results, we randomly tested 35 of insertions and deletions for PCR validation which had passed local *de novo* assembly pipeline. We found that 30 out of 35 (deletion: 18/21, insertion: 12/14) were successfully confirmed and yielded robust products with the expected sizes and positions (Table S12).

### Association of SVs with various genomic landscapes

It is now widely accepted that genetic variant is distributed non-randomly across the genome^49^. It is key to pinpoint the biases of SVs and SV formations for better understanding the feature of pig genome variation. In our study, we analyzed the relationship between SVs and various genome features, including repeat regions, coding regions, recombination hotspots, chromosomal landmarks and GC content.

**Figure 4:**
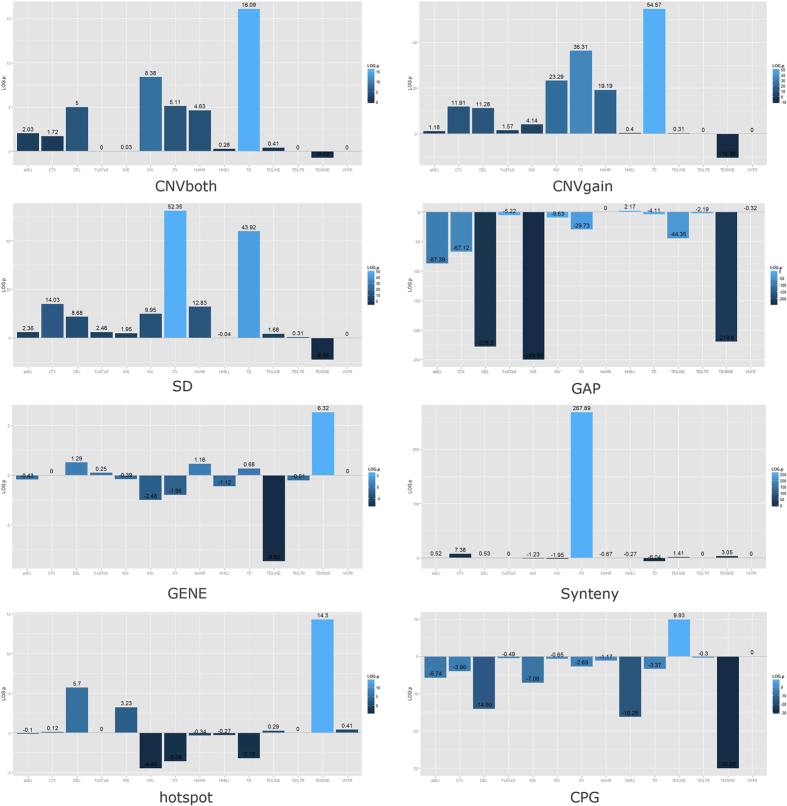
The relationship between various SV events with pig genomic landscapes. Each bar chart respectively shows the relationship between SV events with different genomic landscapes. The absolute value of ordinates represents the absolute value of log p value, and the positive and negative directions of ordinates depict positive and negative relationship between various SV events with corresponding pig genomic landscapes.

As shown in Fig. 4, among all types of SVs, tandem duplication was most significantly associated with genomic SD regions. Regarding SV formational biases, NAHR was significantly enriched within SD regions (3.4 fold, *P* = 1.49 × 10^−13^), which also conformed the previous reports in human studies^50^.

Besides, we observed that almost all SV events are negatively correlated with GAP regions (*P* < 7.79 × 10^−5^). As to coding regions, owing to the importance in the biological function of coding regions, SV events were seldomly associated with coding regions, but negative to LINE elements (0.7-fold, *P* = 2.41 × 10^−9^) and positive to SINE elements (1.2-fold, *P* = 4.8 × 10^−7^). This may be due to coding regions are relatively conserved compared with other regions in genome.

In addition, we also found that chromosomal translocations were significantly enriched (*P* < 4.18 × 10^−8^) in synteny regions, which indicated a very high correlation between synteny block and genomic rearrangements^51^. We correlated SV events with recombination hotspot regions (average recombination rates >1.6) and found that they are typically enriched for deletions (P = 1.98 × 10^−6^), insertions (P = 5.91 × 10^−4^), as well as SINE elements (P = 5.04 × 10–15). This demonstrated that a positive relationship between insertion/deletion mutations occur commonly in recombination hot spots.

Finally, the high GC-rich regions (GC ratios >60%) were found to have a significant negative correlation with most of SVs, such as deletions (P = 8.08 × 10^−15^) and insertions (P = 8.32 × 10^−8^), *etc*. A summary of correlations of SVs and various genomic landscrapes are given in Table S13.

It should be noted that, in SV enrichment analyses related to GC-content regions, we merely focused on those with high GC ratio >60% similar as the criterion of Paul *et al.*^52^. Although Tortereau *et al.*^53^ found that GC-rich sequences strongly associated with high recombination rates in the pig genome, no obvious positive correlation was reflected for those genomic regions where GC content reaches a high level of above 0.55, especially a comparative low recombination rate level could be predicted as the GC content exceeds 60%. Hence, these extremely high GC-enriched regions with low recombination rate could be considered usually more stable than other genomic regions, which also has been mentioned by Mugal *et al.*^54^. This can accordingly explain why SVs exhibited a significant depletion distribution in these high GC-content regions in our study.

### Correlation analyses among various genomic variants

**Figure 5:**
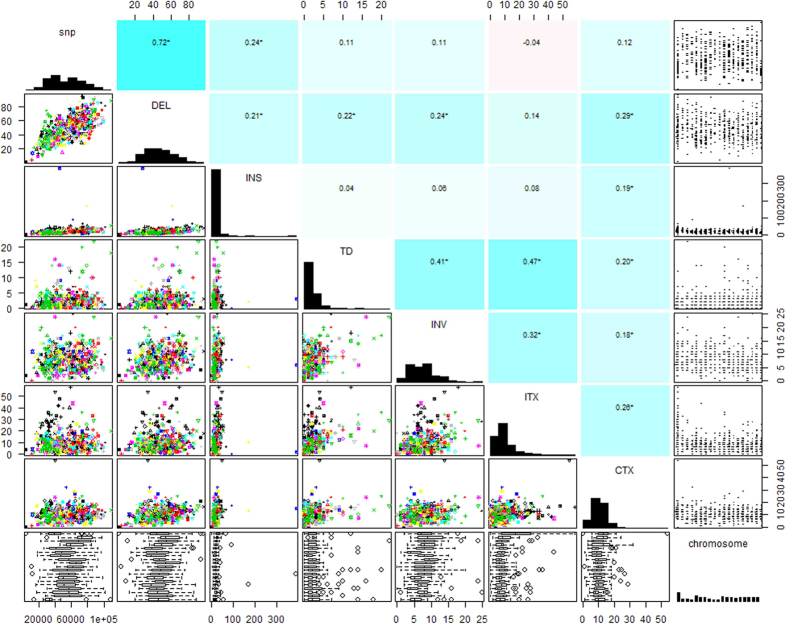
Degree of correlation between distinct variations in the whole genome. The graph that consists of three parts illustrates the degree of correlation between distinct genomic variations in the whole genome. The explicit correlation coefficients between diverse variation are indicated in the upper triangular matrix (numerical value), the lower triangular matrix (scatter diagram), and the diagonal of graph illustrates the specific distribution of SV density. Additionally, the bottom and right-most show the distribution of each SV event among different chromosomes by box plot and scatter plot, respectively.

We found significant correlation between different pair of genomic variants, and the highest correlation was evident (Fig. 5) between deletions and SNPs (r = 0.71, *P* < 2.2 × 10^−16^). Moreover, to further characterize the distribution of SVs, we performed cluster analyses between various variants across different chromosomes. The dendrograms for diverse variations and chromosomes in the resulting heat map (Figure S4) clearly demonstrated the correlation of diverse variations across the genome, which also suggested that deletions and SNPs occurred in a highly consistent fashion across the whole genome.

### Cladistic analyses among individuals from diverse populations

**Figure 6:**
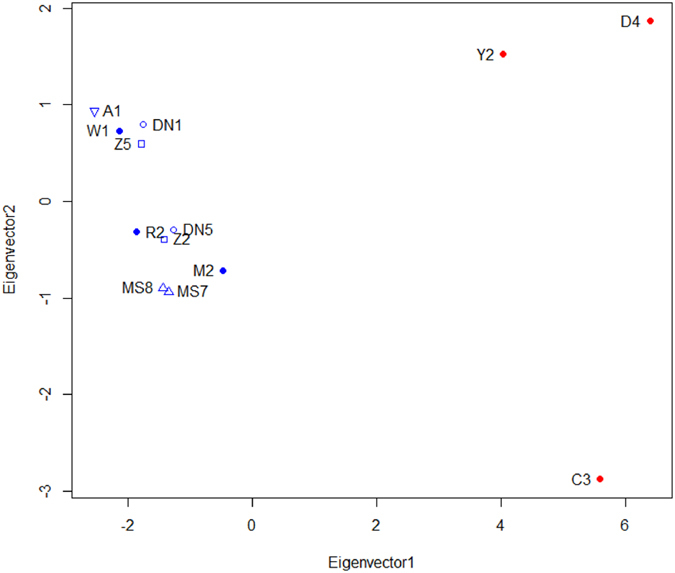
Principal component analyses based on all available SV information. Individuals are plotted according to their coordinates on the biplot of PC1 versus PC2. The blue marker represents the Chinese breeds and the red marker represents the European breeds, the symbols types of these markers represent specific breeds respectively.

We performed principle component analysis (PCA) based on the full set of identified SVs called by DELLY (only DELLY can predict genotypes of corresponding SVs) to detect the genetic structure of individuals surveyed. As shown in Fig. 6, the first two components presented a clear separation between Chinese and European originated individuals. The detected genetic structure constructed herein was consistent with those based on SNPs and other genetic markers^18^. Meawhile, we constructed a NJ phylogenic tree for the 13 individuals based on information of all idetifed SV (Figure S5). The resulting trees reflected the geographic proximity for Chinese pigs and the divergence between Chinese and European pigs, which are consistent with those in other studies^15^,^19^. In addition, each type of SV was employed to perfrom PCA analyses at the whole genome scale (Figures S6–S9), further reflecting the specific genetic structure entailed by different sources of genomic SVs.

### Confirmation of a Chinese population specific SV hotspot using large-scale NGS data

**Figure 7:**
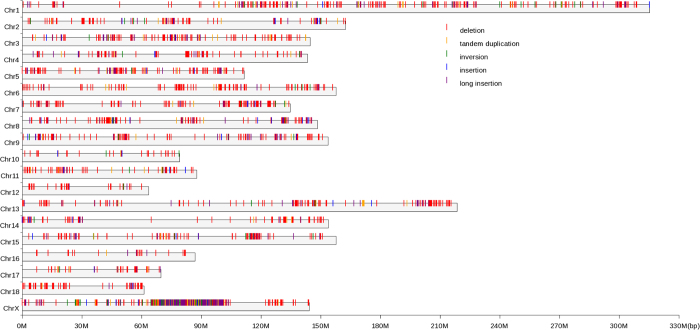
The distribution of the Chinese breeds specific SVs. The SVs that merely appear in Chinese breeds while absent in European population were presented in this study. The gray stripe represents all 19 chromosomes (18 autosomes and X chromosome). The vertical bars indicate different categories of SVs, and the colors red, orange, green, blue, and violet represent deletion, tandem duplication, inversion, insertion, and long insertion, respectively.

To further scrutinize potential SVs strongly contributing to the divergence between Chinese and Europeon breeds, we sifted out 4,071 Chinese originated individual specific SVs (Table S14–S20) from all identified SVs. The distribution of these SVs was also presented in Fig. 7. We processed functional annotation within these possible breed specific SV regions, and found 701 functional gene involved therein. We further performed Gene Ontology (GO) and KEGG analyses for these genes (Tables S21–S24). The GO analyses revealed 95 significant GO terms (P < 0.01), which mainly comprised biological adhesion, biological regulation and immune function. Besides, these genes were also enriched in mTOR signaling pathway (P = 1.90 × 10^−3^) which sensed and integrated a variety of environmental cues to regulate organismal growth and homeostasis, as well as played a role in regulating muscle mass and adipogenesis^55^. The enrichment of specific GO categories and pathways might contribute to the diversities between Chinese and European pig breeds and the changes in patterns of selective constraint in specific traits.

**Figure 8:**
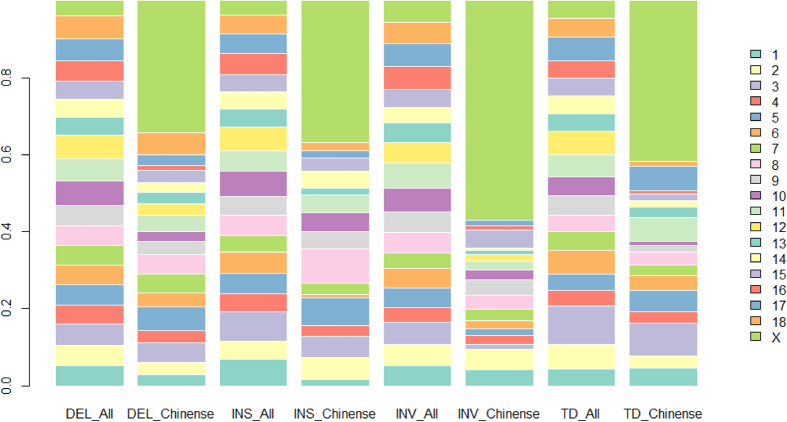
The percentage distribution of the Chinese specific SVs and all detected SVs on chromosomes. Eight bars including four different SVs, and each SV bar respectively represents all or Chinese specific variation. In addition, the chromosomes are indicated by different colors.

Further screening the distributions of these Chinese individual specific SVs, we found each type of these SVs was approximately evenly distributed across the genome except the X chromosome, where an extremely high proportion of SVs (1,577/4,071) was present in contrast to the propotion of SVs (70/672) identified among European individuals (Fig. 8). It is intriguing that among these 1,577 SVs, 1,267 out of them (except translocations) clustered within a 35 Mb region from 65 Mb to 100 Mb, which can be preliminarily speculatd as a SV hotspot region harboured merely in the genomes of Chiense originated individuals.

Note that the Chinese population-specific 35-Mb SV hotspot initially identified herein on the X chromosome was merely speculated based on 13 individuals investigated. To obtain a more convincing evidence of this SV hotspot, we further performed SV detection within this specific region using large-scale NGS data of 111 independent individuals from five different breeds, *i.e.*, two Chinese indigenous breeds including 30 Meishan and 28 Tibetan pig individuals, three most commonly used European breeds including 34 Duroc, 5 Landrace and 14 Large White pig individuals. Amazingly, the SV hotspot was successfully confirmed on the X chromosome of Chinese indigenous breeds while absent in European Population (Figure S10 and Table S25). The results further confirmed the evidence that a 35-Mb SV hotspot specifically occurs on the X chromosome of Chinese originated population.

### Gene contents and evolutionary analyses in the SV hotspot on X Chromosome

In order to pursue more comprehensive understanding of the high divergence of SVs within this region, we further performed SV detections among the genomes of extra 14 wild pig individuals worldwide (NGS data was downloaded from Frantz *et al.*^56^). We found that a high proportion of these Chinese domestic population enriched SVs (~47%–65%) were also present in the Southeast Chinese wild boars. Accordingly, we hypothesized that the SV divergence between Chinese domestic and European pigs within the 35-Mb region could be explained by two possible aspects: Firstly, the findings from Ai *et al.*^19^ demonstrated that the genome sequence of this region in European pigs and some of northern Chinese wild boars was most likely introgressed from an ancient extinct *Sus* species. This could induce exceptionally high divergence (~800 million years) within this SV hotspot region between Chinese domestic pigs and European pigs. Secondly, given the findings aforementioned that merely the Southeast Chinese wild boars out of 14 wild pig individuals shared a very high proportion of SVs within this region, we speculated another introgression fragment from unknown Sus species likely occurred on Chromosome X of Southeast Chinese wild boars after the divergence between Southeast Chinese wild boars and other wild pig populations. The assumption proposed herein should be further confirmed via more comprehensive evidence of porcine demography and evolution which will be pursued in our future endeavors.

Besides, in order to further explore potential biological significance for this SV hotspot region, we further annotated all 132 genes harbored within the region of 65 Mb to 100 Mb on the X chromosome. The identified SVs were mostly overlapped or close (<50 bp) to the regions of these 132 genes (Table S26). We found a suite of promising genes which have been proven associated with fertility in humans, such as, *POF1B* related to premature ovarian failure^57^,^58^, *DIAPH2*^59^ and *RPS4* involved in ovary development. Interestingly, we also found four genes including *ZNF711*, *ATRX*, *SRPX2* and *MAGT1* were reported underlying human mental retardation^60^,^61^,^62^,^63^. Potential roles of these genes on behavioral differences between Chinese and European originated pig breeds will be a follow-up study in future.

In efforts to further investigate the role of the speculated introgression fragment on the X chromosome in the process of gene evolution, we carried out evolutionary analyses within this region. Following the idea of Ai *et al.*^19^, we chose the genome of Phacochoerus africanus (ERR173203) as the outgroup reference and evaluate molecular rates of gene evolution (*Ka*, *Ks* and *Ka/Ks*)^64^ using SNPs called from NGS data of the 111 individuals in Chinese and European domestic populations respectively.

After excluding those no any non-synonymous substitutions among all individuals as well as with uncertain function genes (LOC#########), we finally determined evolutionary rates of 39 genes in Chinese and European pig populations respectively. Thirty-five out of these 39 genes (Table S27) have a significantly different evolutionary rate (P < 0.01) between Chinese and European originated breeds. The resulting *Ka/Ks* clearly reflected the role of speculated introgression fragment in the evolutionary history of Chinese and European domestic pigs. Intriguingly, we also found all these 35 genes with significantly different evolutionary rates between Chinese and European populations have no any nonsynonymous substitutions among all 56 individuals from European breeds investigated. Especially, three genes out of them, *i.e.*, *POF1B, DIAPH2, RPS4*, with significantly higher evolutionary rates in Chinese populations are all involved in the process of ovary development, which may be considered as promising genes contributing to the difference of reproduction ability between the two different sources of populations.

## Discussion

In present study, we firstly identified a Chinese population specific SV hotspot of 65–100 Mb on the X chromosome. We further successfully confirmed this novel finding by large-scale NGS data of 111 individuals from five distinct breeds originated from both China and Europe. Furthermore, we found 35 genes within this region entailed a significant difference of molecular evolutionary rates (*Ka/Ks*) between Chinese indigenous population and three European breeds.

Based on the evidence above, we can logically speculate that Chinese population specific SV hotspot in the 65–100 Mb region on X Chromosome significantly contributes to different evolution rates of function important genes within this region. Furthermore, since these genes, such as *POF1B, DIAPH2* and *RPS4*, are mainly related to reproduction abilities, we also further infer these genes with high frequencies of both nonsynonymous substitutions and SVs in Chinese pig populations can be considered as a key factor explaining the genetic difference of corresponding phenotypes and features between Chinese and European breeds. Potential genetic effects rising from this SV region merit further investigation in the future.

It is also notable that the region identified herein almost overlapped completely with that reported in other two independent studies. Specifically, Songbai *et al.* identified a physical position 40–80 Mb on the X chromosome which involved particularly high Fst estimates between Chinese indigenous and European commercial breeds, reflecting selective sweep signals in Chinese swine populations^65^; Similarly, Chao *et al.* found a remarkable homozygosity region in X Chromosome ranging from 64.7 to 101.2 Mb in Chinese, but not in European breeds^66^. Findings herein as well as from the two other studies further supported that a specific region on X Chromosome can be considered as a hotspot of variants contributing to the genetic difference between Chinese and European breeds. Different from reports elsewhere, the present study firstly pinpointed a substantial SV hotspot region involving a number of genes with nonsynonymous substitutions existing on the X chromosome specifically in Chinese population. This provides a novel evolutionary evidence for understanding phylogenetic relationships among Chinese and European pig breeds.

In our study, we employed various SVs instead of commonly-used SNPs to infer genetic structure of individuls surveyed. We found SVs can clearly detected the difference of genetic background between individuals from Chinese and European originated breeds, which has been widely reported based on traditional genetic markers, *e.g.*, SNPs and microsatellites. This clues us that genome-wide SVs can capture majority of geneic variation and be applied into relevant fields, *e.g.*, evolution genetics and breeding practices, especially under the situation that a huge number of SVs can be readily detected using NGS data.

Inference of the mechanisms of a SV locus is crucial for relating SV surveys to pig genome evolution and population genetics. The nucleotide-resolution SV map developed herein provides an easy way to investigate the processes involved in SV formation. Considering the character of different SV detection algorithm and reliability of the findings, we merely considered the deletion as the target for formation mechanism inference. This is because that the breakpoints of deletion have a high accuracy and the mechanism of deletion more likely to be successfully detected compared with other SVs. We found that the majority patterns of formation mechanism in the pig genome were TEI-SINEs, which was largely consistent with those in humans^37^. It may be because SINEs have relatively short sizes and are more likely neutral compared to others. Different from simple deletion loci, approximately two thousands of complex deletions were inferred as dominating mechanisms of NHEJ and FoSTeS. It would suggest that FoSTeS could generate genomic, genic and exonic complex rearrangements^38^. The effect of such complex SV has been reported in human studies, *e.g.*, the BRCA-1 mutation that associated with breast/ovarian cancer was a deletion/insertion at the same nucleotide position^67^. Roles of these complex structure variations among the pig genome are worthy of in-depth study in future.

We also observed that the number of identified SVs was far less than that of SNPs. This conservative mutation rate of SVs could maintain the fundamental function of the pig genome since SVs have been considered having potentially larger effects than SNPs by changing gene structure and dosage, alternating gene regulation, exposing recessive alleles and other mechanisms^25^,^26^. Furthermore, the most common type of all SVs was deletions, which is consistent with similar studies in other species^27^. This may be due to the existing SV detection methods were prone to deletion identification in contrast to other types of SVs^5^,^24^,^28^.

We found that the highest correlation between deletions and SNPs in this study. This further strengthened the notion that deletion variants and SNPs potentially share similar evolutionary histories^68^. Further characterizing the distribution of SNPs within the SV regions, we found that SNPs were significantly enriched around the SV breakpoints, *i.e.*, the regions closer to SV breakpoints, the more likely SNPs produced. This further supports that SNPs and SVs should share a similar formation mechanism in the genome.

In summary, we herein take into account RP and SR algorithm simultaneously via most popular detection tools to develop a convincing SV porcine map with highest resolution to date. We also employed the *de novo* assembly method for further refining the identified SVs. This improved the SV detection, especially in short reads analysis^69^. Based on such detection strategy, we achieved SV detections with a high accuracy which was also been further confirmed though PCR validation in the analyses. Furthermore, we systematically characterized whole genome SVs and found that SVs are significantly enriched/depleted within various genomic features. We also further exploited formation mechanisms of the majority of identified deletions and unraveled the dominating mechanism of TEI for deletions in the pig genome. To our knowledge, this is the first study focusing on in-depth detecting and characterizing non-CNV SVs at single-nucleotide resolution in the whole genome of pigs. The high resolution SV map constructed and SV-mutational formation mechanisms inferred herein enabled us to further identify functional elements and also advance evolutionary studies in the pig genome.

## Materials and Methods

### Animal collection

The whole procedure for collection of animal samples was carried out in strict accordance with the protocol approved by the Institutional Animal Care and Use Committee (IACUC) of China Agricultural University.

In this study, in an attempt to acquire the comprehensive spectrum and high resolution of SVs in the pig genome, we collected a total of 13 pigs which were from different geographical regions and relatively different in their physiological characteristics. The experimental samples included a Min pig (M2), a Daweizi pig (W1), a Rongchang pig (R2), two unrelated Tibetan pigs (Z2, Z5), two unrelated Diannan small-ear pigs (DN1,DN5), two unrelated Meishan pigs (MS7, MS8) from six Chinese indigenous breeds; a Landrace (C3), a Duroc (D4) and a Yorkshire (Y2) from three European breeds; and one Asia wild boar (A1).

### Sequencing, QC processing, and NGS data processing

Genomic DNA of 13 individuals was extracted from the ear tissue by Qiagen Dneasy Tissue kit (Qiagen, Germany). The quality of all DNA was evaluated by spectrophotometry and agarosegel electrophoresis, and sequenced by Illumina HiSeq 2000 in BGI. Two paired-end DNA libraries were constructed for 13 pig samples and 421.65G bases were generated totally. To ensure quality, we conducted a quality control step. The raw data was modified by following three steps: 1) the reads that contained adapter sequence were deleted; 2) the reads which contained more than 50 percent low quality bases (quality value ≤5) or more than 10 percent N bases were removed; 3) to facilitate better downstream analysis, we carried out homopolymer trimming to 3′ end of fragments and removed the N bases of 3′ end by NGS QC Toolkit^70^. The Raw read depth for each of 13 individuals ranges from 10.41 to 17.42 (Table S28).

As to reference sequence, we selected Duroc sow 2–14 and downloaded its draft genome sequence (*i.e.* Susscrofa 10.2 reference assembly, ftp://ftp.ensembl.org/pub/release-67/fasta/sus_scrofa/dna/) and for the use of sequence alignment and SV discovery.

For obtaining sensitive and accurate results, we further performed extra analytical pipeline as follows: 1) read mapping and local realignment by Samtools^71^ and BWA^72^, and 2) duplicate marking and base quality recalibration by Picard and GATK^73^. Finally, we prepared the analysis-ready reads for SVs discovery and filtering.

### Genome-wide detection and filtering of structural variants

The methods used to detect SVs in this study included Read pair (Breakdancer)^22^, and Split read (Pindel)^23^. In order to ensure the discovery of SVs at high sensitivity and specificity, we also take advantage of a more advanced method that integrates read-pair mapping with split-read refinement (DELLY)^24^. The Breakdancer mainly identified insertions, large deletions (>100 bp), inversions, intra-chromosomal rearrangements and inter-chromosomal translocations based on mapped read pairs; Pindel was adopted to detect breakpoints of short deletions, medium-sized insertions and tandem duplication from paired-end short reads; DELLY is suitable for detecting size-extensive deletion and tandem duplication events as well as balanced rearrangements such as inversions or reciprocal translocations. Then, for merging candidate SV calls to create sensitive set and reducing false positives in SV calling, we integrated and filtered the output of Pindel, Breakdancer and DELLY at the unified standard. It required 90% reciprocal overlap to merge SV calls among different software (except the large insertions and translocation), and only those SVs detected by two or more software were selected into our data set. At last, the final input documents used for downstream analyses contained small insertion (>50 bp, <300 bp), large insertions (>300 bp, <10 Mb)^13^, large deletions (>50 bp, <10 Mb), inversions (<10 Mb), tandem duplications (<10 Mb), intra-chromosomal translocations and inter-chromosomal translocations^74^. Besides, for removing the gender effect on SVs detection, Y chromosome was excluded from our analyses.

### Classification of deletion formation mechanisms

Based on the location of merged deletion calls and corresponding breakpoint features, we can determine the mechanism that is likely to generate the observed deletion by Meerkat^39^. In the Meerkat pipeline, the classification criteria are mainly adopted from Kidd *et al.*^75^. In our study, there is a total of six types of mechanisms to be assigned, *i.e.*, TEI, VNTR, NHEJ, alternative end joining (alt-EJ), NAHR and FoSTeS/MMBIR. Additionally, we also detect seven types of definite complex deletions.

### Deriving breakpoints and computational validation for SVs

We developed a local *de novo* assembly pipeline for refining the SV breakpoints and assessing the validity of SV predictions as follow: 1) Generate assembly contigs of candidate SVs using TIGRA-SV^69^ and CAP pipeline^76^; 2) Extract corresponding local reference sequence of SVs from the reference genome; 3) Align the resulting assembly contigs to the local reference sequence using AGE^77^; 4) Generate possible breakpoints of the SV according to the coordinates from the contig alignment, and the resulting breakpoints with the highest frequency from different contig alignments were determined as the final refined breakpoints for the candidate SV.

### DNA Sequencing validation

We randomly selected some of SVs identified in our study for further technical validation by PCR. PCR primers were designed using Primer5 for amplifying the selected SV breakpoints. The primer pairs were designed ~200 bp long for all SVs, and then the PCRs were run on genomic DNA form corresponding sample genomes. Products of PCR were inserted into pMD18T plasmid for DNA sequencing. At last, after sequencing and alignment, if PCRs yielded products of expected sizes and location, this event was determined as being validated successfully. We designed more than two pairs of primers for each of target SV loci.

### Gene ontology analysis and functional classification

We annotated those Genes specific to Chinese originated pigs by BioMart of Ensembl. All of these genes have been tested for cell component, enrichment of molecule function and biological process in gene ontology (GO) terms in Bioconductor^78^ and pathway analysis in DAVID Bioinformatics Resources 6.7^79^. Taking into account the finite number of pig genes assigned to GO terms and pathway analysis, those human annotated genes which were homologous to pig genes were utilized as the background.

### SNP calling

In order to analyze the correlation of distribution between SVs and SNPs, Samtools, bcftools (distributed with Samtools) and vcfutils.pl (distributed with Samtools) were used to call SNPs. We also ran a self-developed Perl script to filter variants with overall quality score (QUAL) below 20 or genotype quality less than 40, and then employed GATK to discard those SNPs within a 10-bp length window of the chromosomal region wherein the numbers of SNPs more than three. Besides, heterozygous SNPs found on sex chromosomes in male individuals were also removed.

### Enrichment analyses via simulation analyses

Firstly, we constructed three random background sets (except long insertion), which had the same size but different locations compared to the real SV data sets. Secondly, we identified the degree of overlap between different SV events and genome feature regions based on four SV databases respectively. Thirdly we examine the degree of overlap consistency of three random background sets by coefficient of variability (Figure S11). Lastly, we test for significant differences between our real SV events database with respect to the average of three simulated SV events database using Fisher’s exact test.

Repeated signals in the DNA are important to format expression of unique coding sequence files and to process additional essential functions for genome replication and accurate transmission to progeny cells^80^. We performed the association analyses between various SV events and repeat regions in the genome, and all repeat regions that came from our previous studies^81^ based on the same materials, including CNVRs (gain and both) as well as SD regions. We analyzed various SV events with chromosomal landmarks, which including the Gap regions (*Susscrofa* 10.2 reference), coding regions (whole gene regions) and synteny regions that represented human-pig synteny block^82^.

Moreover, considering recombination hotspots were an important genomic feature in linkage analyses, we further analyzed the correlation between SVs and genome wide recombination hotspot regions. These regions were selected out from more recent studies^53^, in which recombination map was constructed using the Illumina PorcineSNP60 BeadChip, based on about two thousand five hundred pigs form Four different pig pedigrees (ILL, UIUC, ROS, USDA).

At last, to better understand the relationship between GC content and various SV events, we download the CpG islands (*Susscrofa* 10.2) form NCBI database. Then we selected the GC-rich regions (CG content >60%) in the genome, and assessed associations between these regions and our SV events. Upon the number of respective type of SVs (by midpoint of SVs) and SNP per 5-Mb window for each individual, we calculated the correlation coefficients between diverse variations in the whole genome.

### The collection and calling SVs of large-scale NGS data for confirming SV hotspots

In order to further confirm the SV hotspot on the X Chromosome, we collected a total of 60 animals for re-sequencing, including 30 Meishan pigs and 30 Durocs. Besides, we also downloaded the publicly available NGS data of 56 pigs (30 Tibetan pigs, 5 Durocs, 6 Landraces, and 14 Yorkshires) from NCBI (SRA065461) and EBI (ERP001813) to increase the statistically accuracy of verification. We performed DNA sequencing, NGS data processing as well as SNP and SV calling for these individuals using the same criteria as aforementioned. The mapped read depth for these 111 individuals ranges from 2.69 to 10.2 (Table S29).

### Calculation for the gene evolutionary rates using large-scale NGS data

Calculating the evolutionary rates of genes was based on the SNP genotypes of 111 individuals from five different pig breeds. We calculated the nonsynonymous and synonymous substitution rates among Chinese breeds and western breeds respectively with the genome sequence of wild boar breed Phacochoerus africanus (ERR173203) as the reference using KaKs_Calculator2.0^83^ based on the γ-MYN method^84^.

### Statistical analysis and figures

The length and density distribution of structural variants were mainly performed with R package and perl module. The correlation analysis of the pigs with SV and SNP and PCA based on SVs were analyzed by R package. Figures were generated by applying R v3.02, in-house perl scripts.

## Additional Information

**How to cite this article**: Zhao, P. *et al.* Structural Variant Detection by Large-scale Sequencing Reveals New Evolutionary Evidence on Breed Divergence between Chinese and European Pigs. *Sci. Rep.* **6**, 18501; doi: 10.1038/srep18501 (2016).

## Supplementary Material

Supplementary Information

Supplementary Table S1

Supplementary Table S2

Supplementary Table S3

Supplementary Table S4

Supplementary Table S5

Supplementary Table S6

Supplementary Table S7

Supplementary Table S8

Supplementary Table S9

Supplementary Table S10

Supplementary Table S11

Supplementary Table S12

Supplementary Table S13

Supplementary Table S14

Supplementary Table S15

Supplementary Table S16

Supplementary Table S17

Supplementary Table S18

Supplementary Table S19

Supplementary Table S20

Supplementary Table S21

Supplementary Table S22

Supplementary Table S23

Supplementary Table S24

Supplementary Table S25

Supplementary Table S26

Supplementary Table S27

Supplementary Table S28

Supplementary Table S29

## Figures and Tables

**Table 1:** The number of structural variants for the 13 analyzed pigs.

Individuals ID	Sample name	Deletion	Short insertion	Long insertion	Tandem duplication	Inversion	Intra-chromosomal translocations	Inter-chromosomal translocations	Total^a^
104	M2	13425	5339	7138	453	3040	1284	8600	39279
105	R2	15123	5230	7496	509	3123	1321	10033	42835
106	Z2	14005	6466	7052	460	3027	1274	8597	40881
107	Z5	14239	7271	7053	451	3021	1266	8595	41896
108	DN1	14397	7647	7096	469	3023	1293	8565	42490
109	DN5	14297	6256	7259	475	3044	1287	8553	41171
110	MS7	13976	6622	7203	487	3004	1266	8876	41434
111	MS8	14010	5695	7244	498	3012	1272	8966	40697
112	W1	14501	7254	7167	456	3054	1307	8690	42429
113	C3	10191	5365	5940	384	2859	1208	6490	32437
114	Y2	10628	4854	5882	374	2882	1207	6480	32307
115	D4	8934	4990	5401	342	2844	1135	5764	29410
116	A1	15310	8244	7527	530	3116	1317	9428	45472
	total	173036	81233	89458	5888	39049	16437	107637	512738

^a^The total number of SV detected for each individual.

**Table 2:** The mechanism statistics for the complex deletions.

Type	Mechanism	Amounts
del_ins^a^	FoSTeS	519
del_ins	NHEJ	1015
del_ins	TEI_complex	75
del_ins	TEI_LINE	80
del_ins	TEI_LTR	5
del_ins	TEI_SINE	118
del_ins	VNTR	4
del_inso^b^	FoSTeS	5
del_inso	NHEJ	1
del_insod^c^	FoSTeS	2
del_insou^d^	FoSTeS	9
del_inssd^e^	FoSTeS	14
del_inssd	NHEJ	3
del_inssu^f^	FoSTeS	7
del_inssu	NHEJ	2
del_invers^g^	FoSTeS	10

Note: The amounts of different complex deletion and their corresponding formation mechanisms.

^a^Deletion with insertion at the break point with unknown source.

^b^Deletion with insertion at the break point, insertion comes from a different chromosome, opposite orientation.

^c^Deletion with insertion at the break point, insertion comes from the same chromosome, opposite orientation and downstream of deletion.

^d^Deletion with insertion at the break point, insertion comes from the same chromosome, opposite orientation and upstream of deletion.

^e^Deletion with insertion at the break point, insertion comes from the same chromosome, same orientation and downstream of deletion.

^f^Deletion with insertion at the break point, insertion comes from the same chromosome, same orientation and upstream of deletion.

^g^Deletion with inversion at the break point, inversion comes from deleted part.
