# Supplementary material for: Structural Variant Detection by Large-scale Sequencing Reveals New Evolutionary Evidence on Breed Divergence between Chinese and European Pigs
Source: Sci Rep. 2016 Jan 5;6:18501. doi: 10.1038/srep18501 (PMC4700453; doi:10.1038/srep18501)
Supplement: Supplementary Information [file srep18501-s1.pdf]

Structural Variant Detection by Large-scale Sequencing Reveals New Evolutionary Evidence on Breed Divergence between Chinese and European Pigs

Pengju Zhao[1*], Junhui Li[1*], Huimin Kang[1], Haifei Wang[1], Ziyao Fan[1], Zongjun Yin[2], Jiafu Wang[3], Qin Zhang[1], Zhiquan Wang[4] and Jian-Feng Liu[1†]

Supplemental Information

Includes:

Supplemental Figure legends (Fig.S1-S11)

Supplemental Figures (Fig.S1-S11)

Supplemental Tables (Tables S1-29)

Figure S legends

Figure S1. Comprehensive structural variation map of 13 pig genomes

Each chromosome bar represents the real location of various SVs for 13 pig genomes. From top to bottom, it represents different individuals of Min pig (M2), Rongchang pig (R2), Tibetan pigs (Z2, Z5), Diannan small-ear pigs (DN1,DN5), Meishan pigs (MS7,MS8), Daweizi pig (W1), Landrace (C3), Duroc (D4), Yorkshire (Y2) and wild boar (A1), respectively. Within each chromosome, the red bar, blue bar, green bar, orange bar, and purple bar respectively represents the deletion, short insertion, inversion, tandem duplication and long insertion.

Figure S2. Formation types of different complex deletion

The red arrow represents deletion fragment, and the blue arrow represents insertion fragment, and the direction of the arrow represents the direction of the sequence. They constitute six kinds of complex SVs: 1) del_inssd: deletion with insertion at the break point, insertion comes from the same chromosome, same orientation and downstream of deletion; 2) del_inssu: deletion with insertion at the break point, insertion comes from the same chromosome, same orientation and upstream of deletion; 3) del_insod: deletion with insertion at the break point, insertion comes from the same chromosome, opposite orientation and downstream of deletion; 4) del_insou: deletion with insertion at the break point, insertion comes from the same chromosome, opposite orientation and upstream of deletion; 5) del_inso: deletion with insertion at the break point, insertion comes from a different chromosome, opposite orientation; 6) del_invers: deletion with inversion at the break point, inversion comes from deleted part.

Figure S3. Local *de novo* assembly pipeline

Five local de novo assembly pipelines were used to further identify the accurate SVs breakpoints. Each local de novo assembly pipeline mainly includes three steps: 1) extract the sample reads form the SVs breakpoints region; 2) de novo assembly based on these extracted sample reads; 3) alignment the de novo assembly contigs with reference genome.

Figure S4. The comparison of correlation coefficients between diverse variations among different chromosomes

The heat map mainly illustrates the correlation coefficients between diverse variations among different chromosomes. The ordinate indicates 19 chromosomes, and the abscissa indicates 10 matched pairs for different types of variations. The darkness of the color indicates the degree of correlation: the darkest red marks the highest degree of correlation, and the darkest blue marks the lowest degree of correlation.

Figure S5. NJ phylogenic tree for the 13 individuals based on all available SV information

Figure S6. Principal component analyses based on deletion information

Individuals are plotted according to their coordinates on the biplot of PC1 versus PC2. The blue marker represents the Chinese breeds and the red marker represents the European breeds, the symbols types of these markers represent specific breeds respectively.

Figure S7. Principal component analyses based on inversion information

Figure S8. Principal component analyses based on tandem duplication

Figure S9. Principal component analyses based on inter-translocation SV information

Figure S10. The distribution of the Chinese breeds specific SVs among 111 individuals

The SVs that merely appear in Chinese breeds while absent in Western population were presented in 58 Chinese indigenous pigs (30 Meishan pigs, 28 Tibetan pigs) and 53 European commercial breeds (34 Durocs, 5 Landraces, 14 Yorkshires). The gray stripe represents all 19 chromosomes (18 autosomes and X chromosome), the vertical bar indicates different categories of SVs. The colors red, orange, and green represent deletion, tandem duplication, and inversion, respectively.

Figure S11. Coefficient of variability for different SV events among three random backgrounds

The SV events were classified by the different color box, the length of the box represents the according range of CV (Coefficient of variability).

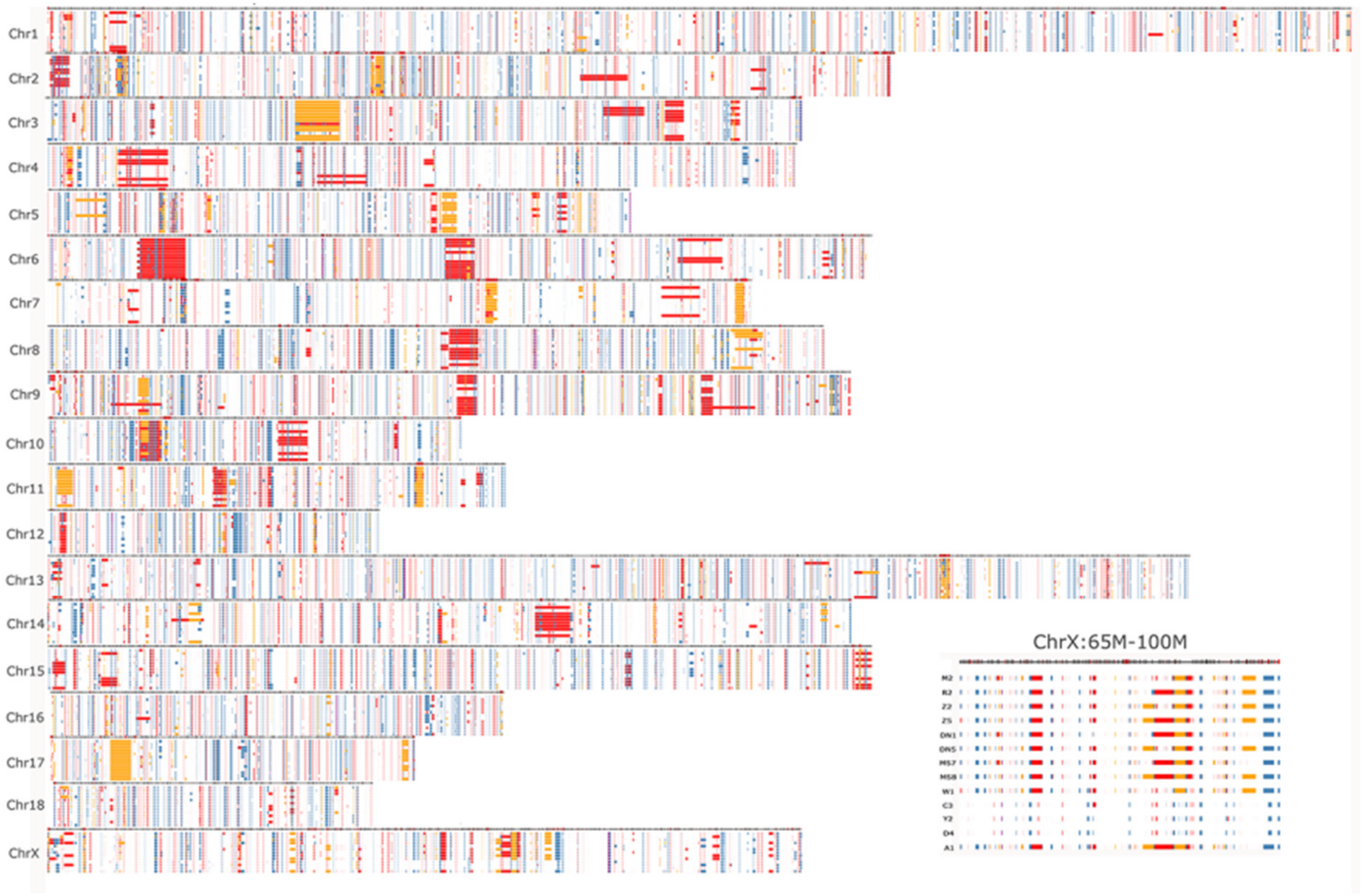

Figure S1. Comprehensive structural variation map of 13 pig genomes

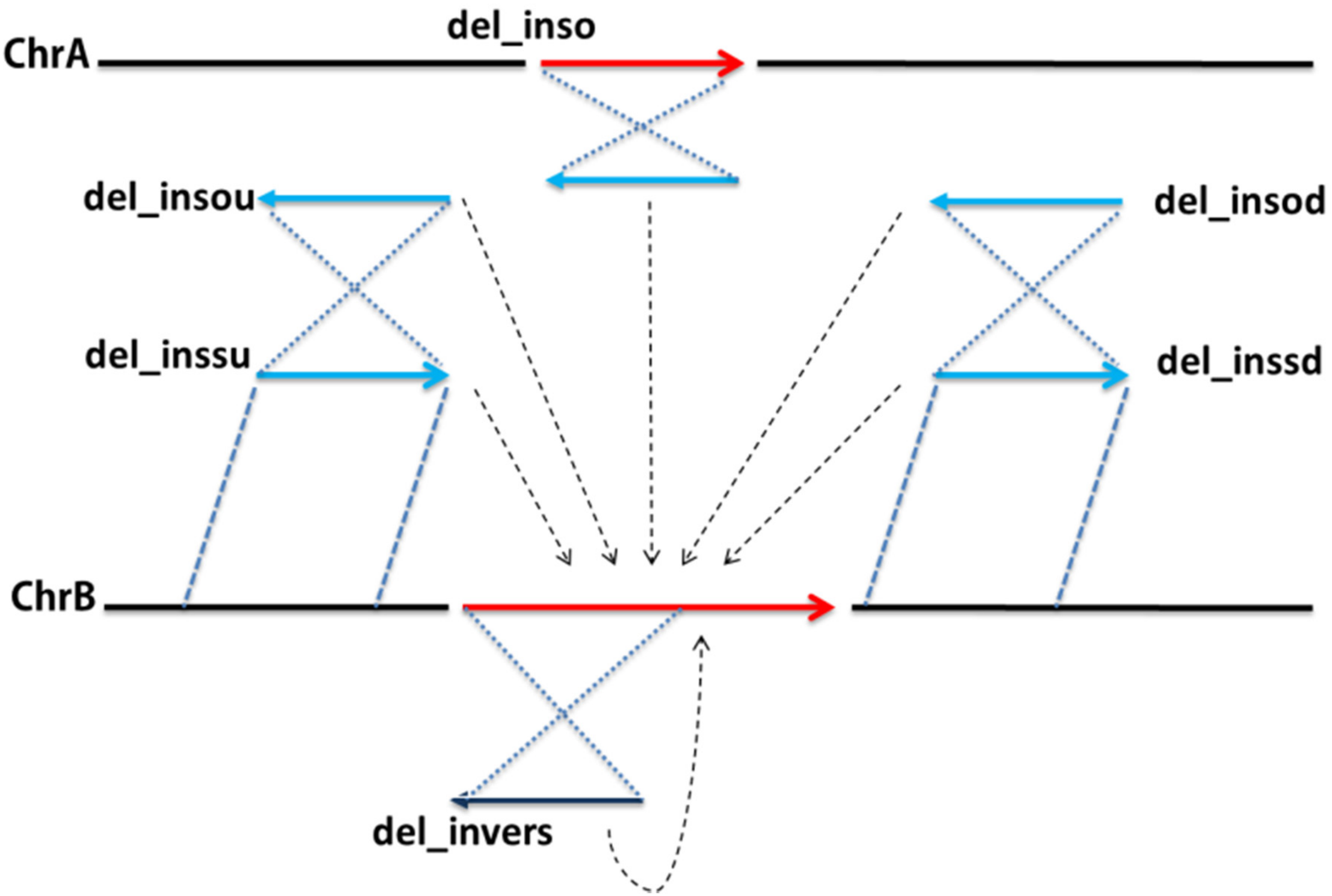

Figure S2. Formation types of different complex deletion

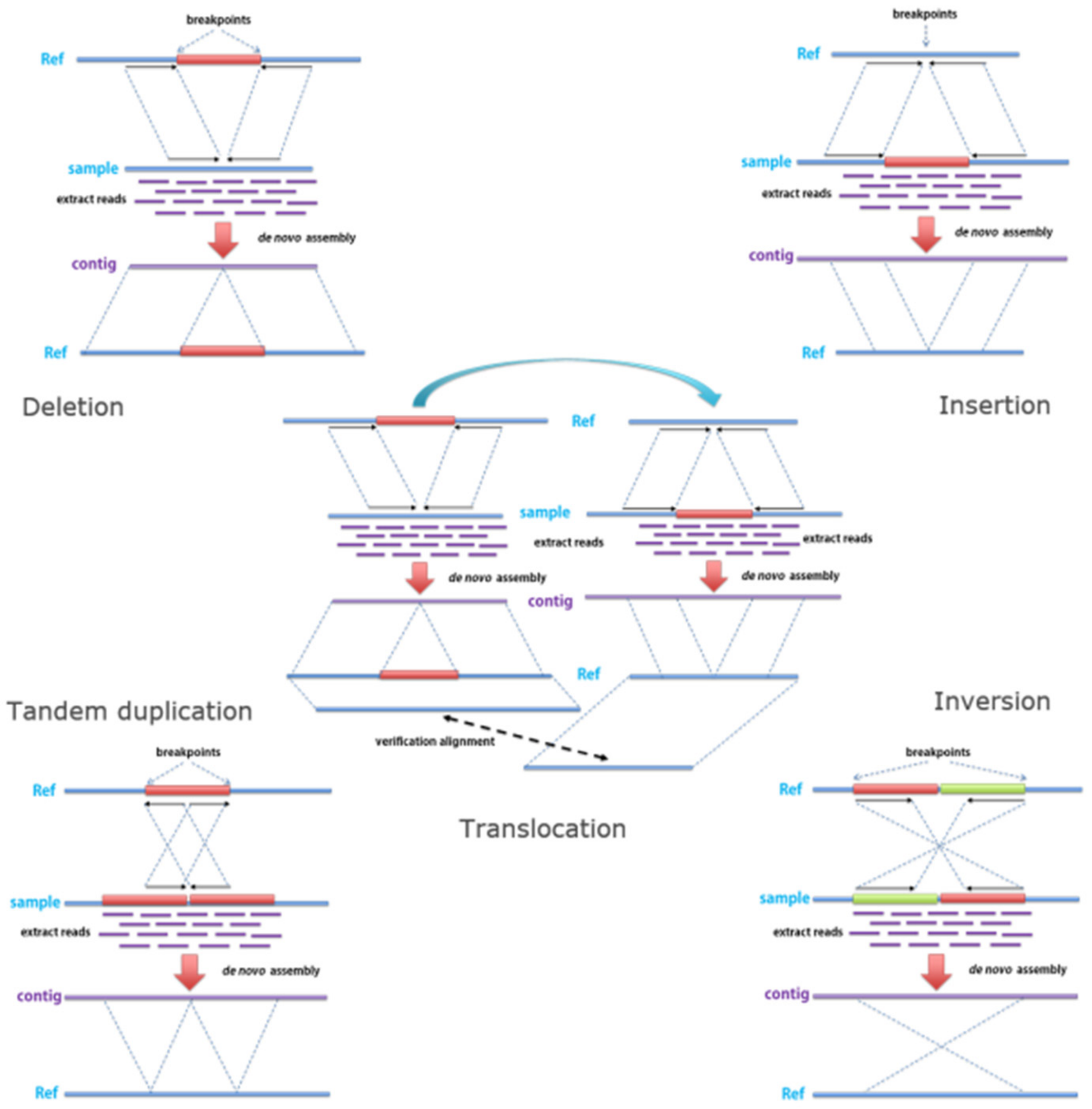

Figure S3. Local *de novo* assembly pipeline

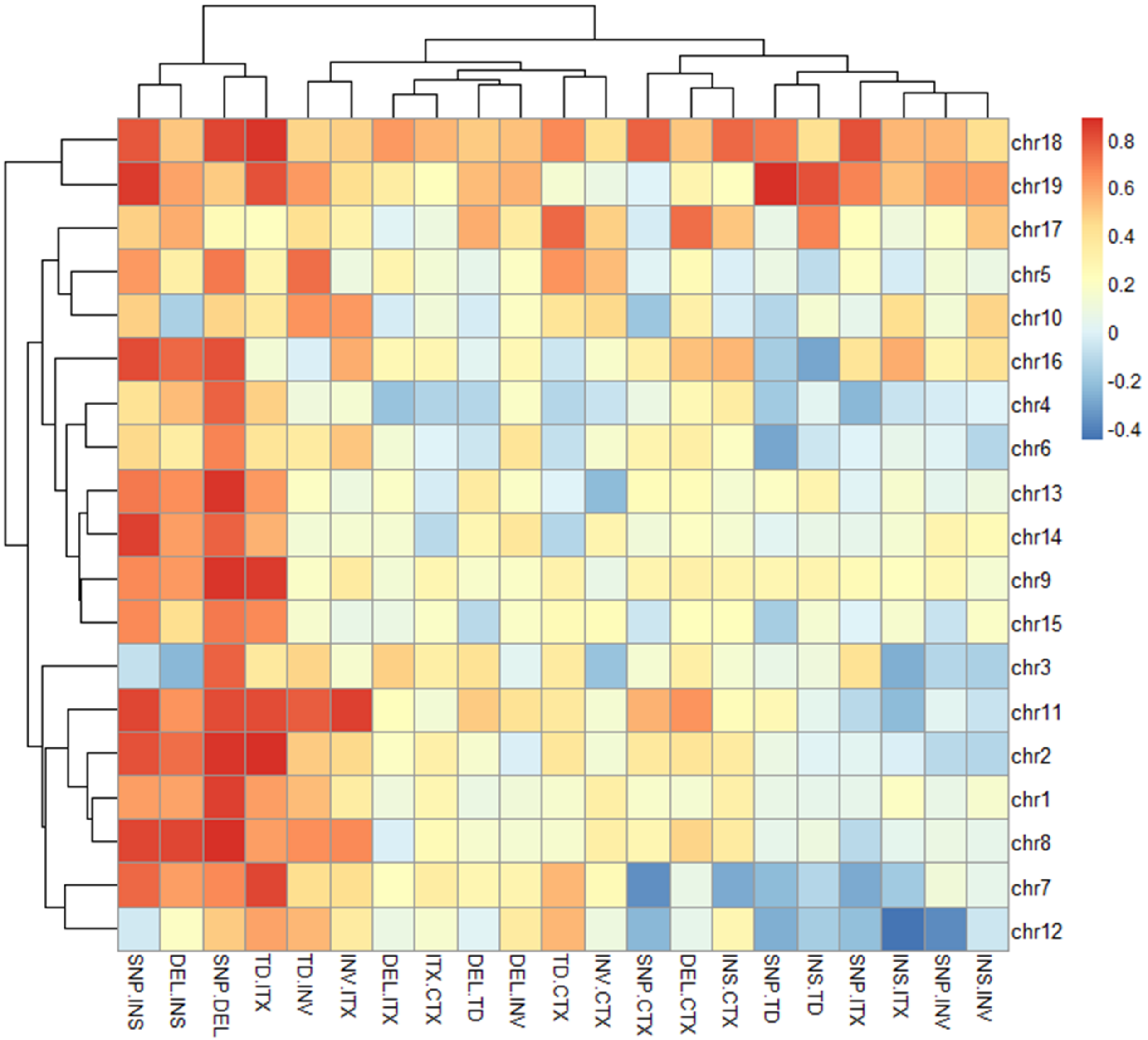

Figure S4. The comparison of correlation coefficients between diverse variations among different chromosomes

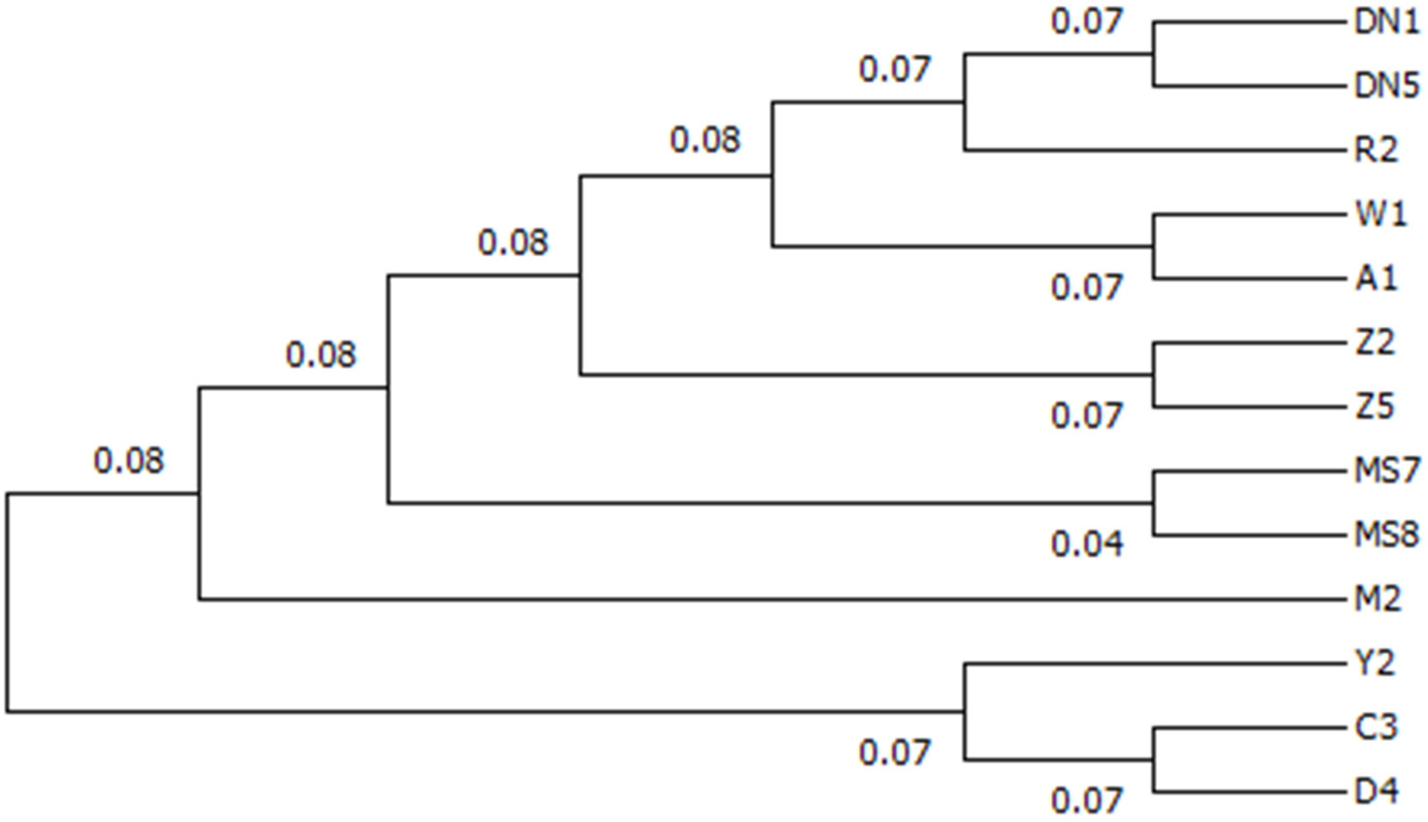

Figure S5. NJ phylogenic tree for the 13 individuals based on all available SV information

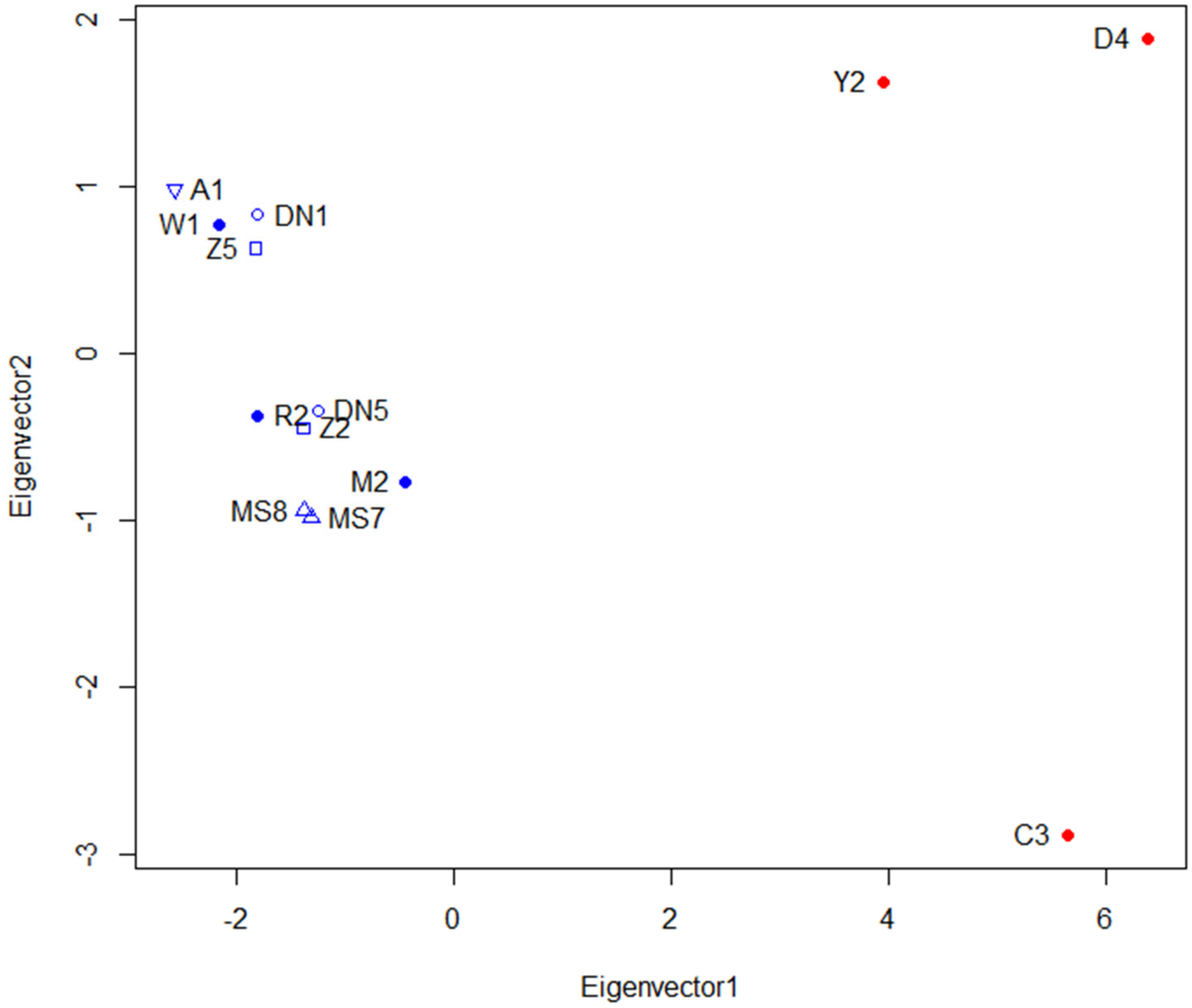

Figure S6. Principal component analyses based on deletion information

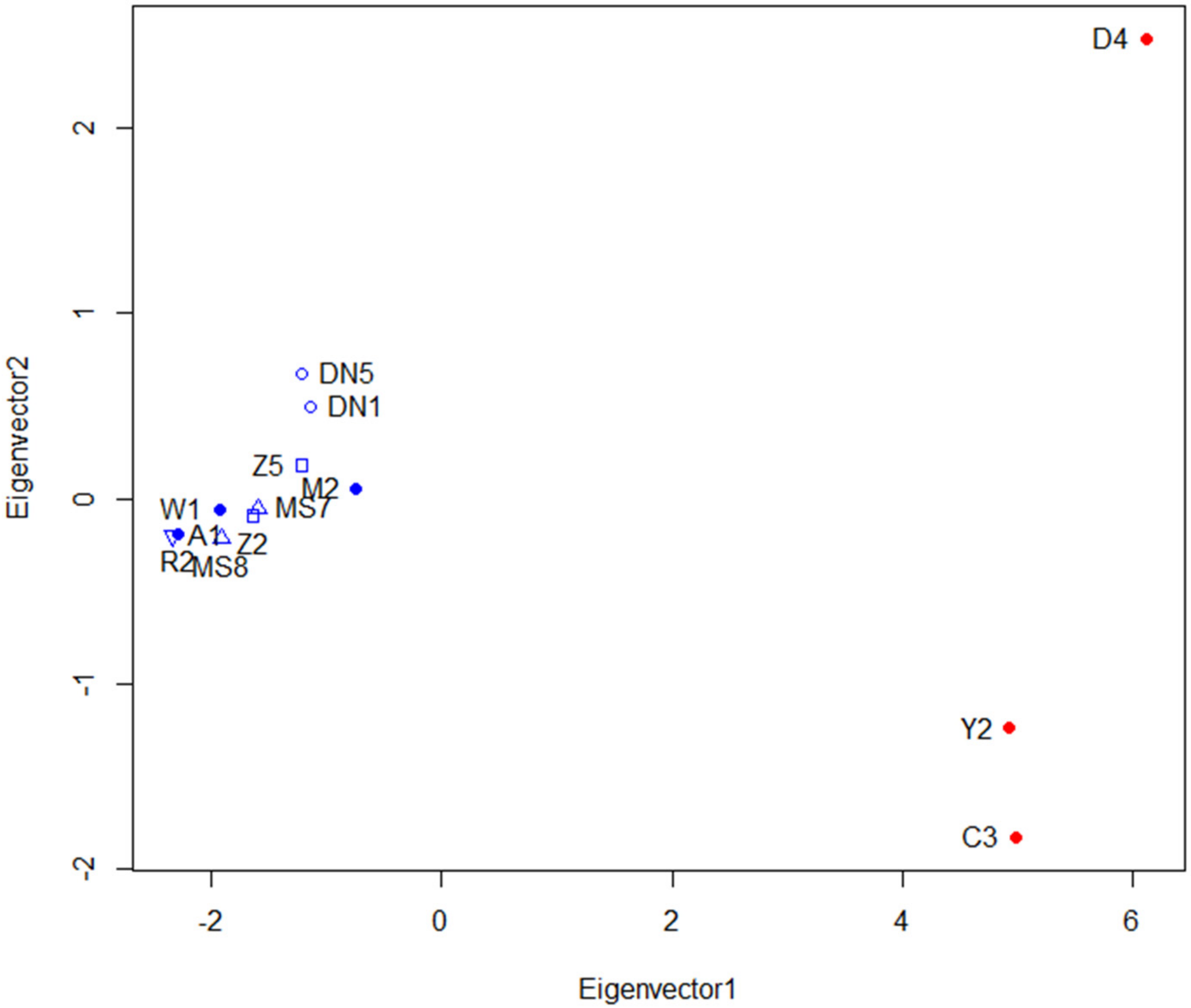

Figure S7. Principal component analyses based on inversion information

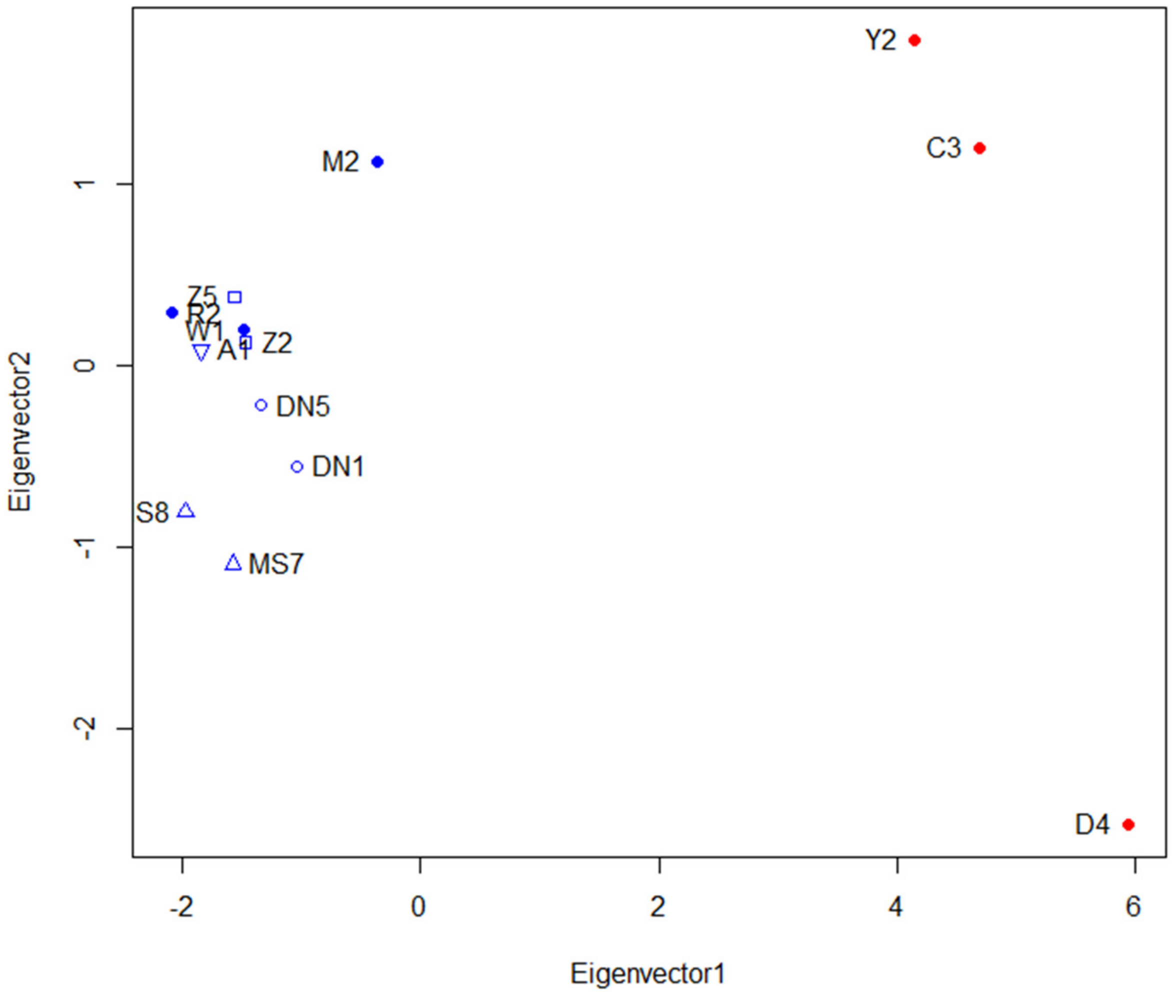

Figure S8. Principal component analyses based on tandem duplication information

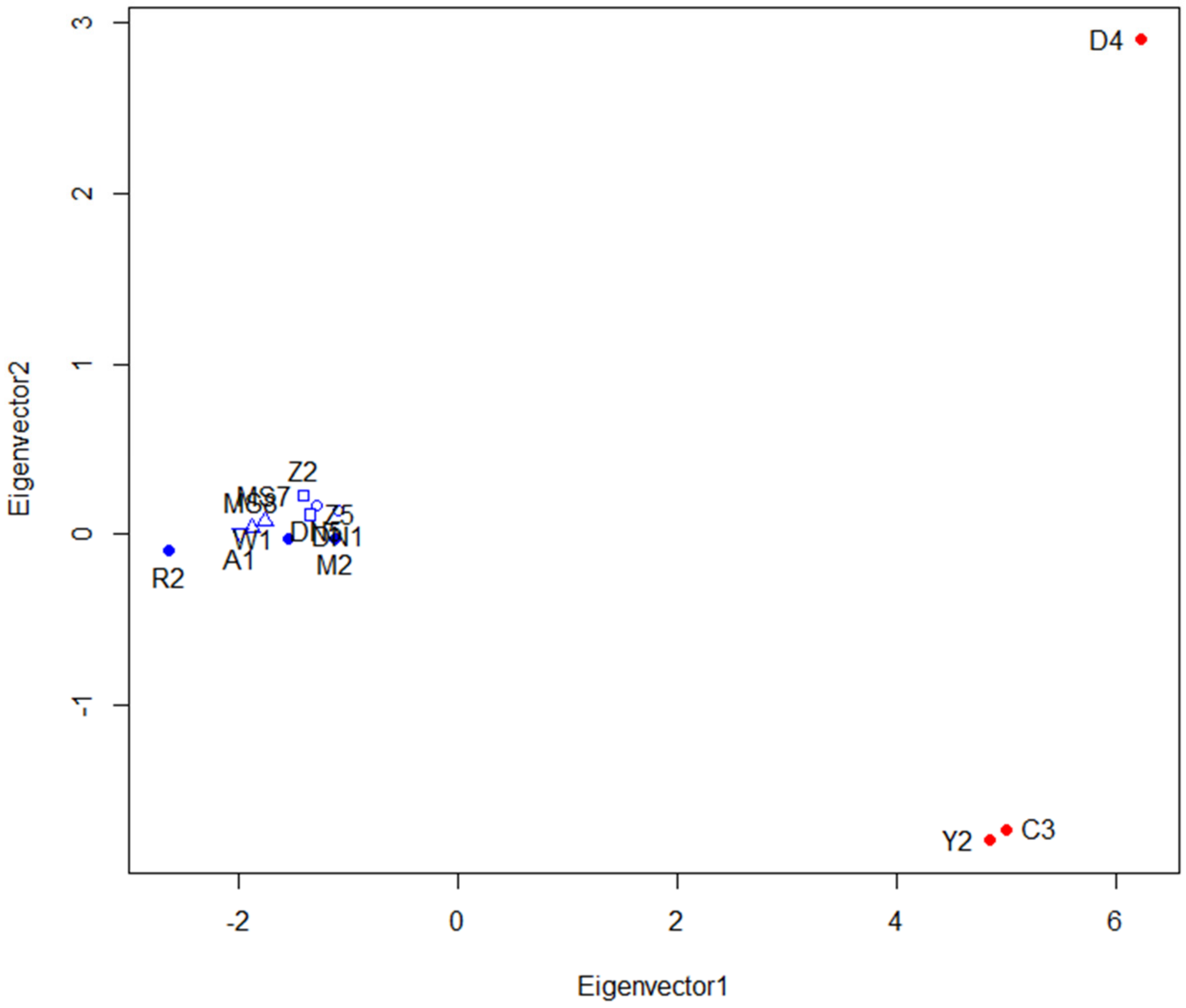

Figure S9. Principal component analyses based on inter-translocation SV information

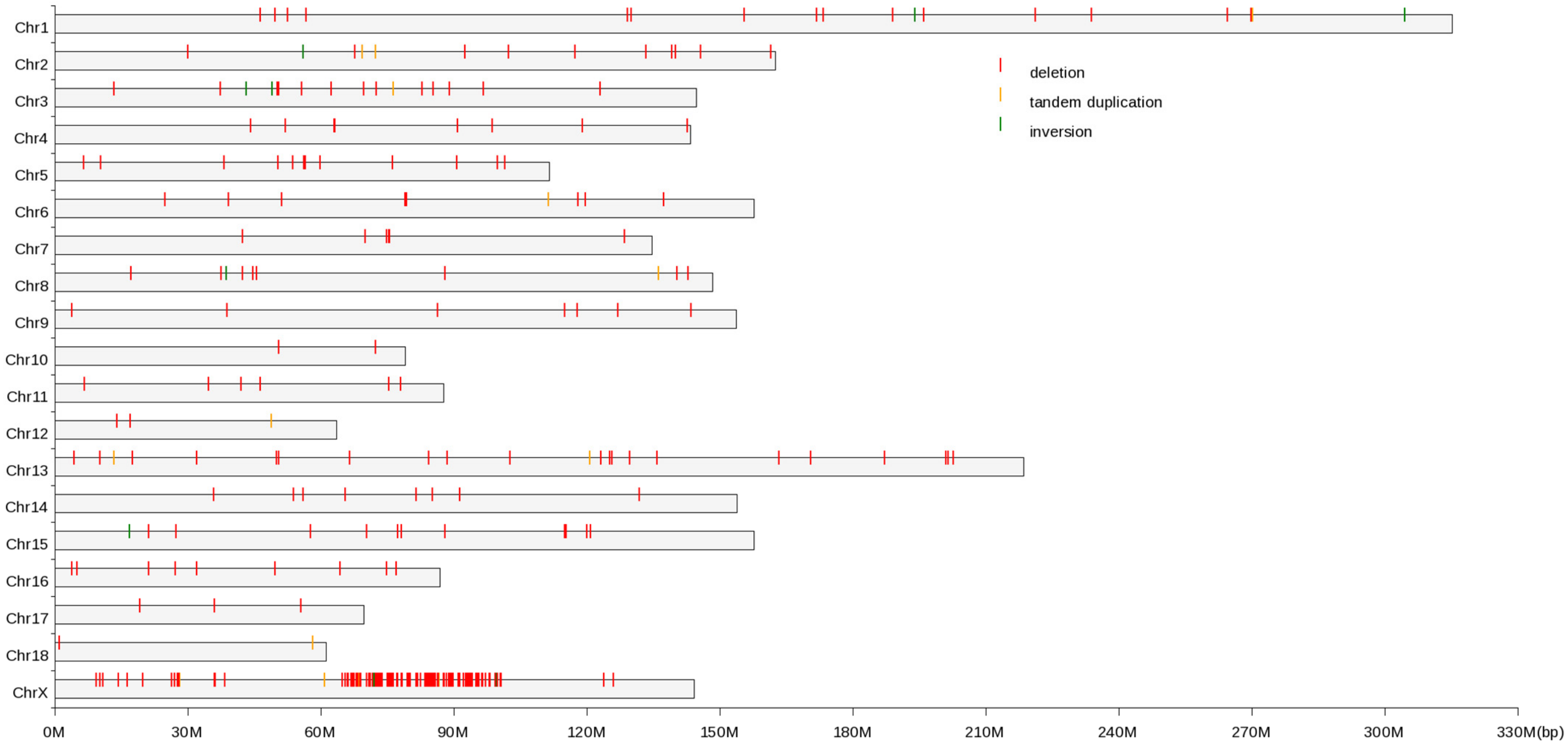

Figure S10. The distribution of the Chinese breeds specific SVs among 111 individuals

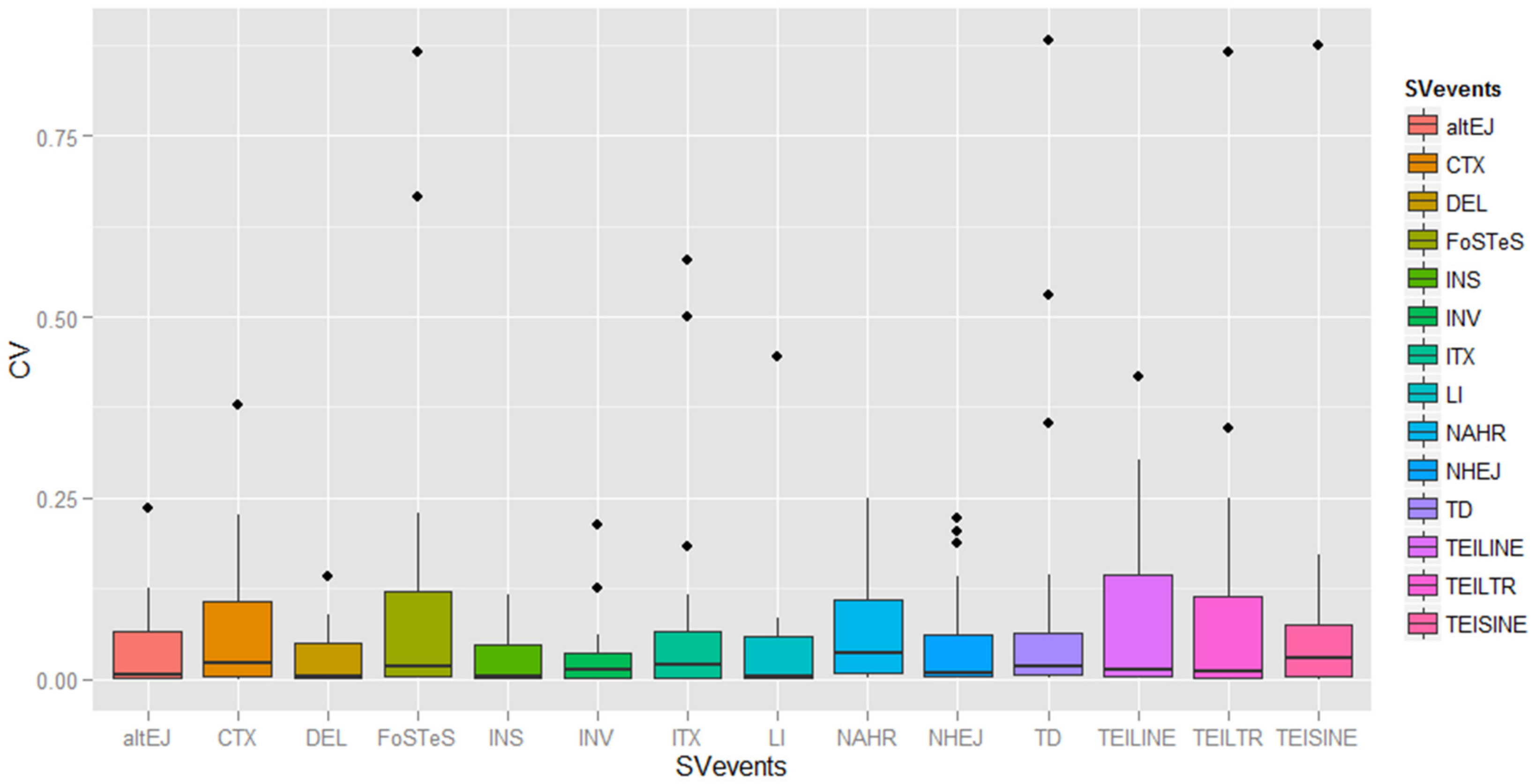

Figure S11. Coefficient of variability for different SV events among three random backgrounds
